# Exploring the putative microRNAs cross-kingdom transfer in *Solanum lycopersicum-Meloidogyne incognita* interactions

**DOI:** 10.3389/fpls.2024.1383986

**Published:** 2024-05-08

**Authors:** Paola Leonetti, Debora Dallera, Davide De Marchi, Pamela Candito, Lorenzo Pasotti, Anca Macovei

**Affiliations:** ^1^ Institute for Sustainable Plant Protection of the National Research Council, Unit of Bari, Bari, Italy; ^2^ Laboratory of Bioinformatics, Mathematical Modelling, and Synthetic Biology, Department of Electrical, Computer and Biomedical Engineering - Centre for Health Technology, University of Pavia, Pavia, Italy; ^3^ Plant Biotechnology Laboratory, Department of Biology and Biotechnology “L. Spallanzani”, University of Pavia, Pavia, Italy

**Keywords:** bioinformatics pipeline, miRNAs, cross-species target prediction, plant-pathogen interaction, tomato, root-knot nematode

## Abstract

**Introduction:**

Plant-pathogen interaction is an inexhaustible source of information on how to sustainably control diseases that negatively affect agricultural production. *Meloidogyne incognita* is a root-knot nematode (RKN), representing a pest for many crops, including tomato (*Solanum lycopersicum*). RKNs are a global threat to agriculture, especially under climate change, and RNA technologies offer a potential alternative to chemical nematicides. While endogenous microRNAs have been identified in both *S. lycopersicum* and *M. incognita*, and their roles have been related to the regulation of developmental changes, no study has investigated the miRNAs cross-kingdom transfer during this interaction.

**Methods:**

Here, we propose a bioinformatics pipeline to highlight potential miRNA-dependent cross-kingdom interactions between tomato and *M. incognita*.

**Results:**

The obtained data show that nematode miRNAs putatively targeting tomato genes are mostly related to detrimental effects on plant development and defense. Similarly, tomato miRNAs putatively targeting *M. incognita* biological processes have negative effects on digestion, mobility, and reproduction. To experimentally test this hypothesis, an *in vitro* feeding assay was carried out using sly-miRNAs selected from the bioinformatics approach. The results show that two tomato miRNAs (sly-miRNA156a, sly-miR169f) soaked by juvenile larvae (J2s) affected their ability to infect plant roots and form galls. This was also coupled with a significant downregulation of predicted target genes (*Minc11367, Minc00111*), as revealed by a qRT-PCR analysis.

**Discussions:**

Therefore, the current study expands the knowledge related to the cross-kingdom miRNAs involvement in host-parasite interactions and could pave the way for the application of exogenous plant miRNAs as tools to control nematode infection.

## 1 Introduction

Root-knot nematodes (RKNs) are pathogens that attack many economically important crops, negatively impacting crop yield, quality, and subsequently food security (Kaloshian and Teixeira, 2019). Damage caused by RKNs has been estimated to range between 80 - 157 billion $US per year, although this evaluation may be largely underestimated (Palomares-Rius et al., 2021). Among the RKNs, members of the *Meloidogyne* genus are the most widespread and have a broad range of hosts. These parasites can penetrate host roots and induce the formation of specialized feeding structures (root galls), which supply the resources required for nematode development. The formation of root galls is highly damaging because they affect the plants’ ability to uptake water and nutrients (Singh et al., 2015). Tomato (*Solanum lycopersicum* L.), one of the most important and extensively grown horticultural crops in the Mediterranean region (EUROSTAT, 2021), is the preferential host for many *Meloidogyne* species. Yield losses due to *M. incognita* RKNs can range between 25-100% (Seid et al., 2015). Control methods generally include the use of chemical fumigants or nematicides, but since the ban of chemicals with a broad action on non-target organisms, emerging RKN populations continue to bypass plant host defenses. Therefore, alternative approaches (e.g., eco-friendly fumigants, bio-control agents) that can stimulate plant defense mechanisms, are needed to control their spread (Leonetti and Molinari, 2020). Currently, much focus is given to the use of RNA interference (RNAi) as an eco-friendly strategy (Banerjee et al., 2018; Banakar et al., 2020; Iqbal et al., 2020), as demonstrated for fungal small RNAs that suppress plant immunity by hijacking host RNAi pathways (Weiberg et al., 2013; Hua et al., 2018). Examples of cross-kingdom RNAi during plant-pathogen interactions are also available. For instance, the *Botrytis cinerea* sRNA produced by Dicer-like (DCL) proteins can target and silence *DCL* genes in Arabidopsis with subsequent effects on fungal pathogenicity and growth (Wang et al., 2016). Other examples include the transfer of ds-siRNA and miRNAs from plants to Coleoptera species, with consequences on gene transcription and insect growth (Zhang L. L. et al., 2019; Kleaveland, 2023). Additionally, miRNAs are being investigated as important regulatory actors in the plant-nematode interaction (Jaubert-Possamai et al., 2019), supporting the development of novel tools to advance modern agriculture.

As small, evolutionary conserved, and generally non-coding RNA molecules, microRNAs finely regulate gene expression being involved in developmental and stress responses. In plants, miRNAs achieve their function through perfect or near-perfect complementarity to target mRNAs, while in animals three types of miRNA-target interactions are recognized: (1) partial binding, mainly to the seed region; (2) complete or near-complete binding that enables AGO-mediated endonucleolytic cleavage of the target; and (3) extended/bulged binding to the seed region which specifies target-directed miRNA degradation (Kleaveland, 2023). When addressing host-parasite interactions, the involvement of miRNAs and long non-coding RNAs in the relation between *S. lycopersicum* and *M. incognita*, has been investigated (Kaur et al., 2017; Yang et al., 2020, 2022). While Zhang Y. et al. (2016) focused mainly on the high-throughput identification and annotation of miRNAs in *M. incognita*, Kaur et al. (2017) directed their attention to the identification of tomato miRNAs in susceptible plants during nematode infection. However, no study has investigated the miRNAs cross-species potential during this interaction. Several works have studied miRNAs transfer to other organisms along with possible regulatory functions (LaMonte et al., 2012; Zhang et al., 2012; Cheng et al., 2013; Zhu et al., 2017; Avsar et al., 2020; Cai et al., 2021), making it reasonable to hypothesize that this exchange may be well-represented during plant-parasite interactions, and that this can be exploited as an alternative tool to sustainably fight pathogens (Gualtieri et al., 2020; Rabuma et al., 2022). So far, the miRNA cross-kingdom transfer ability has been demonstrated in several plant-pathogen interactions, like *Gossypium hirsutum*–*Verticillum dahliae* (Zhang H. et al., 2016), *Triticum aestivum*–*Puccinia striiformis* (Wang et al., 2017), and *Arabidopsis thaliana*–*Plutella xylostella* (Zhang L. L. et al., 2019).

In the current study, we propose a bioinformatics pipeline to investigate the putative effects of cross-species miRNAs transfer during the interaction between *S. lycopersicum* - *M. incognita*. Specific miRNAs and transcripts from the two species have been retrieved from public databases and used to predict candidate targets in a cross-kingdom manner, based on different miRNA-mRNA hybridization rules. Biological processes of Gene Ontology significantly affected by the target genes were identified and examples of bidirectional cross-targeting predictive miRNAs, with potentially interesting applications to fight disease development, are provided. Additionally, three tomato miRNAs (sly-miRNA 156a, sly-miRNA166b, sly-miRNA169f) were selected for *in vitro* validation studies on juvenile *M. incognita* larvae (J2s) infecting the roots of susceptible tomato plants.

## 2 Materials and methods

The bioinformatics workflow adopted in this work includes the following steps: (1) miRNA and transcript sequence collection from public data, (2) cross-kingdom miRNA target prediction by two different procedures, and (3) analysis of target genes and enriched biological processes resulting from the predicted miRNA-target pairs. Subsequently, experimental methods for the *in vitro* evaluation of the effect of selected *S. lycopersicum* miRNAs on *M. incognita* larvae are provided.

### 2.1 Datasets

A collection of *S. lycopersicum* miRNAs (sly-miRNAs) was obtained by merging the entries found in public databases and literature. The miRbase repository (Kozomara et al., 2019) included 147 sly-miRNAs, of which 137 were unique. The sly-miRNA list reported by Kaur et al. (2017) was added, including 136 sequences (56 miRNAs classified as conserved or variants, and 60 as novel miRNAs), which account for 70 unique miRNAs after duplicate removal. Thus, a total of 207 sly-miRNAs (obtained from the two merged lists), with 185 unique sequences, represent the final collection used for tomato miRNAs. The *S. lycopersicum* transcript dataset (ITAG 2.4.v) was retrieved from psRNATarget (Dai et al., 2018) and included 34,725 transcripts, covering a large part of the tomato annotated genome (37,872 genes) available on NCBI.

Since *M. incognita* miRNAs (min-miRNAs) are not included in public databases, only literature works were used to define this collection. The list of Zhang Y. et al. (2016) included 144 min-miRNAs (38 classified as conserved and 106 as novel miRNAs), which accounts for 63 unique miRNAs after duplicate removal. The list of Wang et al. (2015) included 102 min-miRNAs, corresponding to 70 unique sequences, of which about 40% is composed of conserved miRNAs. From the 133 min-miRNAs obtained by merging the two lists, 126 were unique and represented the final collection of RKN miRNAs. The *M. incognita* transcript and CDS datasets were retrieved from the V2 genome assembly (Blanc-Mathieu et al., 2017), available at the INRAE *Meloidogyne* Genomic Resources website (https://*meloidogyne*.inrae.fr), which includes 43,718 transcript and CDS sequences. The *M. incognita* 3’UTRome was obtained by processing the transcripts and CDS lists above with a custom Python (v3.8) script, resulting in 20,201 sequences because not all the full transcripts have an annotated 3’UTR.

### 2.2 miRNA target prediction

The cross-kingdom search of miRNA targets in *S. lycopersicum* and *M. incognita* transcriptome was performed using the psRNATarget (https://www.zhaolab.org/psRNATarget/) and RNAhybrid (https://bibiserv.cebitec.uni-bielefeld.de/rnahybrid/) tools, respectively, previously proposed for miRNA target prediction in plants (Dai et al., 2018) and animals (Kruger and Rehmsmeier, 2006). The collections of *M. incognita* and *S. lycopersicum* miRNAs were used as input for both tools, together with the transcriptome of the target organism (tomato and nematode, respectively). The two tools have been validated on intra-kingdom miRNA-target data in their original publications. However, it is worth mentioning that no large-scale validation is available for cross-kingdom predictions because of the very low number of validated miRNA-target pairs. Even though no gold standard tool or set of rules has been defined for cross-kingdom miRNA interactions, we assumed that such regulations follow the rules of the host organism, as it was assumed in other works (Shu et al., 2015; Chin et al., 2016; Zhang H. et al., 2016; Hou et al., 2018; Zhao et al., 2018; Bellato et al., 2019), thus motivating the use of plant- and animal-specific prediction tools. Consistent with previous in silico and *in vivo* studies (Liu et al., 2017; Li Z. et al., 2018; Lukasik et al., 2018; Mal et al., 2018; Wang et al., 2018), instead of restricting the search to the 3’UTRome, reported to be the preferential target of endogenous miRNA regulation in animals (Bartel, 2004), the full transcript sequences were used to search for targets in the RKN transcriptome. For the reasons above, the miRNA-target pairs found by the two tools represent putative regulations that may occur in nature, even though other herein neglected biological factors could play important roles.

RNAhybrid and psRNATarget were both run by setting a maximum of 50 targets per miRNA as previously done (Zhang H. et al., 2016; Bellato et al., 2019), to obtain a balanced set of miRNA-target pairs to be analyzed downstream. RNAhybrid was run using a Minimum Free Energy (MFE) threshold of -25 kcal/mol, corresponding to the upper bound of many experimentally found miRNA-target pairs (Zhang H. et al., 2016). Putative targets were ranked based on their MFE value (lowest to highest) for further selection of a subset of these genes, as required in the downstream steps (see 2.3). psRNATarget was run with an Expectation value of 3, corresponding to a slightly relaxed threshold in terms of prediction results stringency, as reported by Dai and Zhao (2011). Default values were used for the other psRNATarget parameters, as previously done in other studies (Bellato et al., 2019): Penalty for G:U pair = 0.5, Penalty for other mismatches = 1, Extra weight in seed region = 1.5, Seed region = 2-13 nucleotides, Mismatches allowed in seed region = 0, HSP size = 19.

In addition to the procedure described above (indicated as *cross-kingdom hybridization*), we followed a second search method that does not assume any hybridization rule in cross-kingdom interaction. This alternative method (indicated as *seed region-based search*) was carried out to find sly-miRNA targets in *M. incognita* transcriptome and included the following steps: (1) intra-kingdom hybridization (i.e., min-miRNAs *vs*. RKN transcriptome via RNA hybrid); (2) selection of all the sly-miRNAs that share a seed region (nucleotides 2-7 of the miRNA) with the collection of min-miRNAs; (3) association of the selected sly-miRNAs to the targets of the min-miRNAs with the same seed region. Location (CDS or UTRs) of the predicted miRNA binding and sequence similarity outside the seed region were also recorded to support further selection steps.

### 2.3 Biological process analysis

Statistically over-represented Gene Ontology (GO) terms in the Biological Process (BP) category were computed via enrichment analysis using the predicted miRNA targets obtained above. Duplicate genes in the target list were removed before analysis. The ClueGO application (v2.5.6) (Bindea et al., 2009) of Cytoscape (v3.7.3) (Shannon et al., 2003) was used as a GO analysis tool. A right-sided hypergeometric test with the Benjamini-Hochberg correction for multiple hypothesis testing and a P-value cutoff of 0.05 was used.

For *M. incognita* enrichment analysis, an *ad hoc* construction of the GO library was necessary for ClueGO analyses, as this organism was not previously included in this tool. To this aim, the GO biological processes list reported by Somvanshi et al. (2018) was used as a source, converted into a ClueGO-compatible format using a custom Python script, and integrated into ClueGO. In total, 5,508 and 6,098 unique GO terms in the BP category were present for *S. lycopersicum* and *M. incognita*, respectively. The BPs presented in this work after enrichment analysis refer to the most relevant term of the functionally grouped term networks provided by ClueGO. The number of input genes in enrichment analysis was set to obtain a comparable number of relevant terms between plant and RKN.

To further analyze the obtained biological processes, individual genes in the miRNA target list associated with the process were considered, and their function was searched in the literature. For *S. lycopersicum*, gene information was retrieved from the ITAG 2.4 annotations available in Phytozome v.12 (DOE JGI, https://jgi.doe.gov/more-intuitive-phytozome-interface/). For *M. incognita*, orthologous genes from other nematodes (mainly *C. elegans*) were obtained using WormBase ParaSite (https://parasite.wormbase.org/index.html) (Howe et al., 2017). This approach was considered because specific information on RKN genes is not directly available in public online resources. Finally, a subset of BP terms from Somvanshi et al. (2018) related to nematode development was selected and used to filter the sly-miRNA targets in the RKN transcriptome.

### 2.4 *In vitro* interaction assays of sly-miRNAs soaked by *M. incognita* juvenile larvae

For the *in vitro* interaction assay, sly-miR166b, sly-miR169f, and sly-miR156a were selected taking into account the bioinformatics data obtained from the cross-kingdom hybridization and the seed region-based search approach. These microRNAs were obtained from Invitrogen™ Custom Primer Service (BMR Genomics, Padova, Italy) and the relative sequences are given: sly-miR166b (5’-UCGGACCAGGCUUCAUUCCCC-3’, STAR strand GGAAUGUUGUCUGGCUCGAGG), sly-miR156a (5’-UUGACAGAAGAUAGAGAGCAC-3’, STAR strand GCUCUCUAUGCUUCUGUCAU) sly-miR169f (5’-UAGGCGUUGUCUGAGGCUAAC-3’, STAR strand AUCCGUUACUGAGGAACCGAUAG).

Susceptible tomato seedlings (Roma cv.) and axenic cultures of phytoparasitic nematodes were prepared as described by Molinari and Miacola (1997). Freshly hatched *M. incognita* J2s (infective second-stage juveniles) larvae were used for soaking experiments, following a modified protocol (Danchin et al., 2013; Tan et al., 2013). About 8,000 J2s were soaked for 24 hours in a 40µl final volume of mineral water containing different solutions: (1) a siRNA designed to have no similarity in the *M. incognita* genome (Dalzell et al., 2010) defined as negative control solution (C-J2); and (2) three different solutions of 0.05 mg/ml sly-miRNAs corresponding to each tested tomato miRNA. J2s from each soaking experiment were washed twice with water by centrifugation at 10,000 g for 3 min, and re-suspended in 100 µl of water. Subsequently, the miRNA-soaked J2s were observed using a Leica M125 stereomicroscope (Leica Microsystem S.r.l, Buccinasco, Milano, Italy) to confirm their vitality and split in two Eppendorf tubes: one used for infection assay and another used for RNA extraction (frozen at -80°C).

For the interaction assay, the sly-miRNAs soaked J2 larvae (approx. 50 J2s/root apex) were loaded in wells placed on agar plates at 0.1 mm from the tomato root. Each plate contained three susceptible tomato seedlings, growth in axenic conditions. The infection was observed for six weeks, during which the penetration in the roots was evaluated in terms of gradual enlargements of the root tip caused by rapid cell division and proliferation. The giant cell induction and galls formation was monitored using a modified LEICA Software image analysis (unpublished), and the following scoring system was used: “+” ranging between 0-30%, “++” ranging between 31-60%, and “+++” ranging between 61-90% (Melillo et al., 2006; Cabrera et al., 2015). The parameters referring to both root enlargements and formation of galls (91-100%), were counted and scored as the number of symptoms normalized to the number of roots in which the phenomena were observed. All experiments were conducted in triplicates coming from two independent trials.

### 2.5 RNA extraction and qRT-PCR analysis

RNA was extracted using the TRIzol Reagent (Invitrogen, CA, USA) method, as indicated by the manufacturer. For this, 500 J2s soaked for 24 h in the control solution (C-J2) or the respective sly-miRNAs (miR156a-J2, miR169f-J2) solutions, were used. For the reverse transcription, 1 μg of total RNA was used along with the QuantiTect Reverse Transcription Kit (QIAGEN S.r.l, Milano) following the manufacturer’s instructions.

The qRT-PCR reactions were carried out using the StepOnePlus (Applied Biosystems, Life Technologies, Zurich, Switzerland) system and assembled in a reaction with 1.5 μl cDNA, 10 μl SYBR^®^ Select Master Mix (Applied Biosystem, Life Technologies, Zurich, Switzerland), 0.2 μl each of 100 μM of forward and reverse primers, and RNAse free water to 20 μl (total volume). Thermocycling was carried out with one cycle at 95°C for 5 min, followed by 40 cycles of 95°C for 45 s and 58°C for 1 min and 72°C for 45 s. The dissociation curve of the final products was checked to ascertain the presence of a single amplification product. The relative quantification was carried out using the 18S ribosomal RNA gene (NCBI accession HE667742.1, WormBase accession Minc3s09153g42974) as a reference gene. The *Minc11367* (WormBase Accession Minc3s00025g01614) gene, was selected and tested as a putative target for sly-miR156a, while *Minc00111* (WormBase Accession Minc3s00001g00015) was selected as a putative target of sly-miR169f. Oligonucleotide sequences to amplify the genes of interest were designed with Primer3Plus (https://primer3plus.com) and further validated through the online software Oligo Analyzer (https://eu.idtdna.com/calc/analyzer). All the oligonucleotide sequences are provided in **Supplementary Table 1**. The ΔΔCt method was used to quantify gene expression. The reactions were performed in triplicate samples of each cDNA and using two independent replicates.

### 2.6 Statistical analysis

Results of experimental data are shown as mean ± standard deviation (SD) obtained from two independent experiments each with three replicates. Statistical analyses were conducted using the two-way analysis of variance (ANOVA), along with the heteroscedastic Student’s *t*-test (where *, *P* ≤ 0.05; **, *P* ≤ 0.01), available within the Microsoft Excel package.

## 3 Results and discussion

### 3.1 Overview of the bioinformatics pipeline

**Figure 1:**
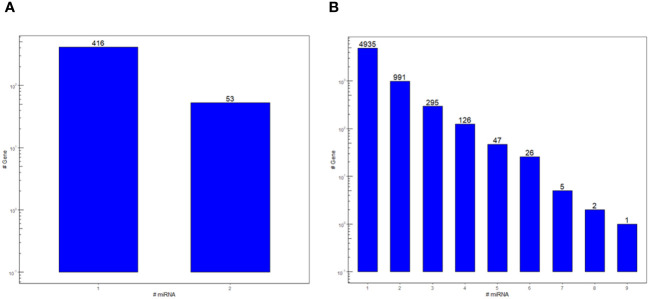
Count of the *S. lycopersicum* **(A)** and *M. incognita* **(B)** miRNA targets in the cross-kingdom hybridization bioinformatic pipeline run on the two species. Bars represent the number of genes that are putatively targeted by one or more miRNA.

In this work, a bidirectional bioinformatics workflow was carried out to predict miRNAs cross-kingdom potential in the host-parasite interaction between tomato and RKNs. These predictions are based on sequence complementarity between miRNAs and cross-species targets, as well as sequence similarity between tomato and nematode miRNAs. A total of 523 miRNA-target pairs were found in the cross-kingdom hybridization between *S. lycopersicum* transcripts and min-miRNAs, corresponding to 469 unique genes and 105 unique min-miRNAs (**Supplementary Dataset 1**). The majority of genes were targeted by a single miRNA, but putative targeting by two miRNAs occurred as well (**Figure 1A**). All the target genes were adopted for subsequent enrichment analysis that yielded 43 biological processes (BPs), divided into 10 functional groups, as described below in Section 3.2. Conversely, 8,926 transcripts corresponding to 6,428 unique genes were found in *M. incognita* by cross-kingdom hybridization with the sly-miRNAs (**Supplementary Dataset 2**). Each gene was putatively targeted by one or more miRNAs (**Figure 1B**). Based on the MFE of the targets, the 290 top-ranked genes were used for the enrichment analysis that resulted in 86 BPs in the 10 functional groups, described further in Section 3.3.

The seed region-based search resulted in the identification of 7 sly-miRNAs having homologies with 9 min-miRNAs; using these sly-miRNAs, a total of 450 putative target genes were identified in *M. incognita* (**Supplementary Dataset 3**).

### 3.2 *M. incognita* miRNAs predicted to target tomato genes may inhibit plant development

**Figure 2:**
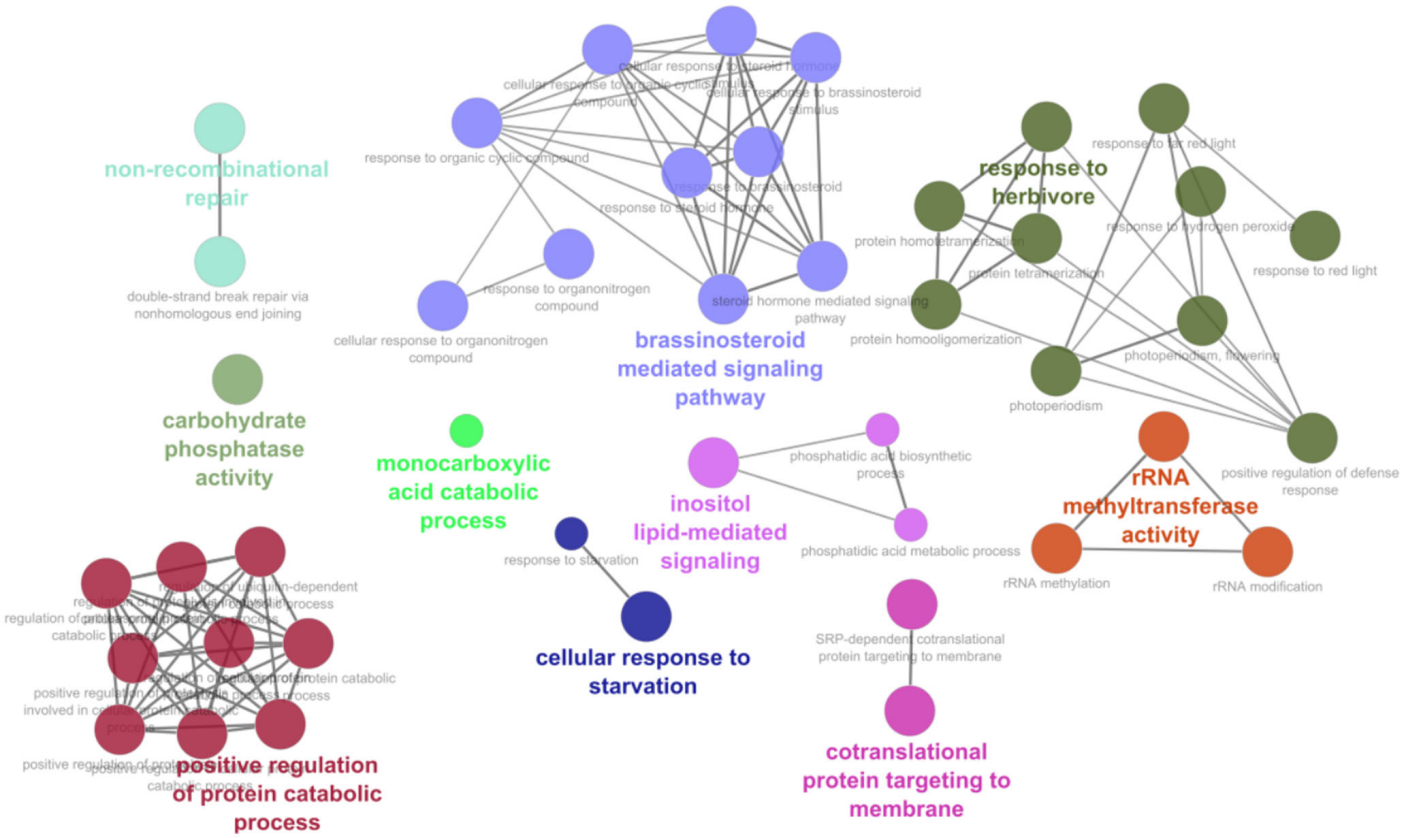
Biological processes and functional network clusters resulting from enrichment analysis carried out using the *M. incognita* miRNA putatively targeting *S. lycopersicum* mRNAs. The most relevant terms of the functional groups are highlighted.

To analyze the obtained datasets, we first looked at the potential effects that min-miRNAs would have on the development of tomato plants providing discussions based on the function of putatively targeted genes. The biological processes found to be enriched among the putative cross-species targets of RKN miRNAs against *S. lycopersicum* transcripts are reported in **Supplementary Dataset 4** and graphically represented in **Figure 2**. These processes clustered in several major GO terms and the most abundant networks included brassinosteroid-mediated signaling pathway, response to herbivores, and positive regulation of protein catabolic process. Other processes like inositol-lipid mediated signaling, co-translational protein targeting to membrane, non-recombinant repair, rRNA methyl transferase, and cellular response to starvation, were less abundant. Considering the min-miRNAs putatively targeting genes in the tomato dataset, the enriched terms list includes 26 miRNAs and 46 unique targets, with the most interesting ones being summarized in **Table 1**. Within the brassinosteroid-mediated signaling pathway, different phosphatases, phosphodiesterase, and proteases were predicted to be targeted by NOVEL-18-1, min_miRNA6, and min_miRNA98. Many protease gene families are well-known to be involved in plant immune responses (Balakireva and Zamyatnin, 2018). For instance, aspartyl proteases are specifically linked to systemic acquired resistance (SAR) induced in response to local infections (Breitenbach et al., 2014; Molinari et al., 2014; Molinari and Leonetti, 2019). Other miRNAs, like NOVEL-8, miR-50, and min_miRNA29, are predicted to target genes playing important roles in the response to herbivores. The S-receptor kinase-like genes, aside from being involved in stress responses and host-pathogen defense, have also important functions in cell signaling and development (Takasaki et al., 2000; Becraft, 2002; Pastuglia et al., 2002). Similarly, genes involved in the cuticle and cell wall development and composition (e.g., sterols, mannans) represent important defense lines against many types of pathogens (Wang et al., 2013; Kazan and Gardiner, 2017). Other min-miRNAs (min_miRNA206, NOVEL-18-1) were predicted to target different E3 ubiquitin-protein ligases in tomatoes; these proteins play important roles in the regulation of cell homeostasis and thus they are key regulators of plant growth and stress responses (Liu et al., 2021). Importantly, they are involved in the regulation of plant innate immunity (Duplan and Rivas, 2014).

**Table 1:** List of representative *M. incognita* miRNAs putatively targeting genes in *S. lycopersicum*, based on bioinformatics cross-kingdom predictions.

GO_ID	GO Term	*M. incognita* miRNA	*S. lycopersicum* Gene Accession	Gene Name
**GO:0009742**	Brassinosteroid mediated signaling pathway	NOVEL-18-1	Solyc06g073960	Calcineurin-like phosphoesterase domain, apaH type
		min_miRNA6	Solyc06g074000	Aspartyl protease
		min_miRNA98	Solyc09g074320	Serine/threonine-protein phosphatase BSL1-related
**GO:0080027**	Response to herbivore	NOVEL-8	Solyc09g008790	serine/threonine-protein kinase SRPK3
		miR-50, miR-50_1	Solyc09g009010	Glucomannan 4-beta-mannosyltransferase
		min_miRNA29	Solyc09g009040	Delta(14)-sterol reductase/Sterol C14-reductase
**GO:0045732**	Positive regulation of protein catabolic process	NOVEL-22-1	Solyc02g069230	RBR family ring finger and IBR domain-containing
		min_miRNA206	Solyc06g073340	E3 ubiquitin-protein ligase ARI10-related
		NOVEL-18-1	Solyc08g005150	E3 ubiquitin-protein ligase RNF14
**GO:0008649**	rRNA methyltransferase activity	NOVEL-44	Solyc01g100430	18S rRNA (adenine(1779)-N(6)/adenine(1780)-N(6))-dimethyltransferase
		NOVEL-6-1	Solyc11g005580	16S rRNA (cytosine(1402)-N(4))-methyltransferase
		miR-76, miR-76_1	Solyc11g020870	Metal dependent hydrolase-related
**GO:0048017**	Inositol lipid-mediated signaling	miR-87	Solyc01g065740	F-box domain
		NOVEL-10-1	Solyc01g066050	RNA polymerase II-associated protein 3
		min_miRNA37	Solyc01g100020	Phospholipase D P2
		min_miRNA206	Solyc06g051720	GDSL esterase/lipase CPRD49
**GO:0006613**	Cotranslational protein targeting to membrane	NOVEL-35	Solyc03g093970	Signal recognition particle subunit SRP68
		miR-76, miR-76_1	Solyc03g116810	Signal recognition particle subunit SRP54
**GO:0072329**	Monocarboxylic acid catabolic process	miR-34_1	Solyc08g005610	Abscisic acid 8’-hydroxylase 1-related
		min_miRNA112 NOVEL-28	Solyc10g008110	Acyl-coenzyme A oxidase-like protein
**GO:0000726**	Non-recombinational repair	NOVEL-12	Solyc01g091350	ATP-dependent DNA helicase 2 subunit 2 (XRCC5, KU80, G22P2)
		miR-87	Solyc01g091370	AT hook motif DNA-binding family protein-related
		let-7	Solyc02g093330	nuclear pore complex protein Nup98-Nup96
		NOVEL-16-1	Solyc02g093690	ATP synthase mitochondrial F1 complex assembly factor 2 (ATPeAF2, ATPAF2, ATP12)
**GO:0009267**	Cellular response to starvation	min_miRNA109	Solyc01g090890	Xenotropic and polytropic retrovirus receptor 1-related, SPX domain-containing protein
		min_miRNA151	Solyc02g037510	Solute carrier family 7 (SLC7A2, ATRC2)
		NOVEL-37	Solyc09g014790	VHS domain containing protein family
**GO:0019203**	Carbohydrate phosphatase activity	miR-81 min_miRNA112	Solyc07g062140 Solyc07g062410	Trehalose-p6-phosphate synthase (TPS)
		min_miRNA206	Solyc01g006740 Solyc10g081660	Sucrose-phosphate phosphatase (SPP)

Gene Ontology (GO) ID and terminology are provided along with gene names and corresponding accessions.

Similarly, miRNAs (e.g., NOVEL-44, NOVEL-6-1, miR-76) affecting translational regulation can play critical roles in the plant defense against pathogen infection. In addition to the essential role of DNA repair in maintaining genome stability, recent works are discussing the involvement of DNA repair proteins in plant-pathogen interactions and SAR (Fu and Dong, 2013; Camborde et al., 2019). Moreover, the miRNA-mediated control over DNA damage responses is starting to gain more interest from both an endogenous (Gualtieri et al., 2021; Macovei et al., 2021) and cross-kingdom fashion (Bellato et al., 2019). An interesting finding in this sense is the case of let-7, one of the most abundant miRNAs found in nematodes, putatively targeting the nuclear pore complex protein Nup98-Nup96 in tomatoes. This complex plays a role in nuclear-cytoplasmic trafficking and mRNA export, being involved in several important biological events such as mitotic checkpoints. Nup98-deficient mutants share pleiotropic phenotypes (decreased root elongation, accelerated floral transition, reduced fertility, and robustness), indicating that it has a critical role in plant development (Parry, 2013). 

Other miRNAs worth mentioning are miR-81, min_miRNA112, and min_miRNA206 predicted to putatively target genes involved in sugar metabolism, like sucrose-phosphate phosphatase (SPP) and trehalose-p6-phosphate synthase (TPS). SSP is an important regulator of carbon partitioning and loss-of-function of this gene leads to altered carbohydrate distribution resulting in a reduced growth rate (Chen et al., 2005, 2008). TPS leads to the formation of the trehalose-6-phosphate (T6P), a metabolic intermediate acting as a signaling molecule that regulates sugar metabolism (Paul, 2008), and its silencing in the tomato caused dysfunction in ROS accumulation and decreased expression of genes responsible for defense against pathogenic infections (Suárez et al., 2008).

To conclude, the prediction analyses show that the tomato genes putatively targeted by *M. incognita* miRNAs have essential roles in plant development and stress response, and their silencing can have negative repercussions for the plant. This is in agreement with the parasitic relation between the two organisms, where *M. incognita* tries to hijack the plant systems to promote its development.

### 3.3 sly-miRNAs predicted to target *M. incognita* genes may have detrimental effects on nematode development

**Figure 3:**
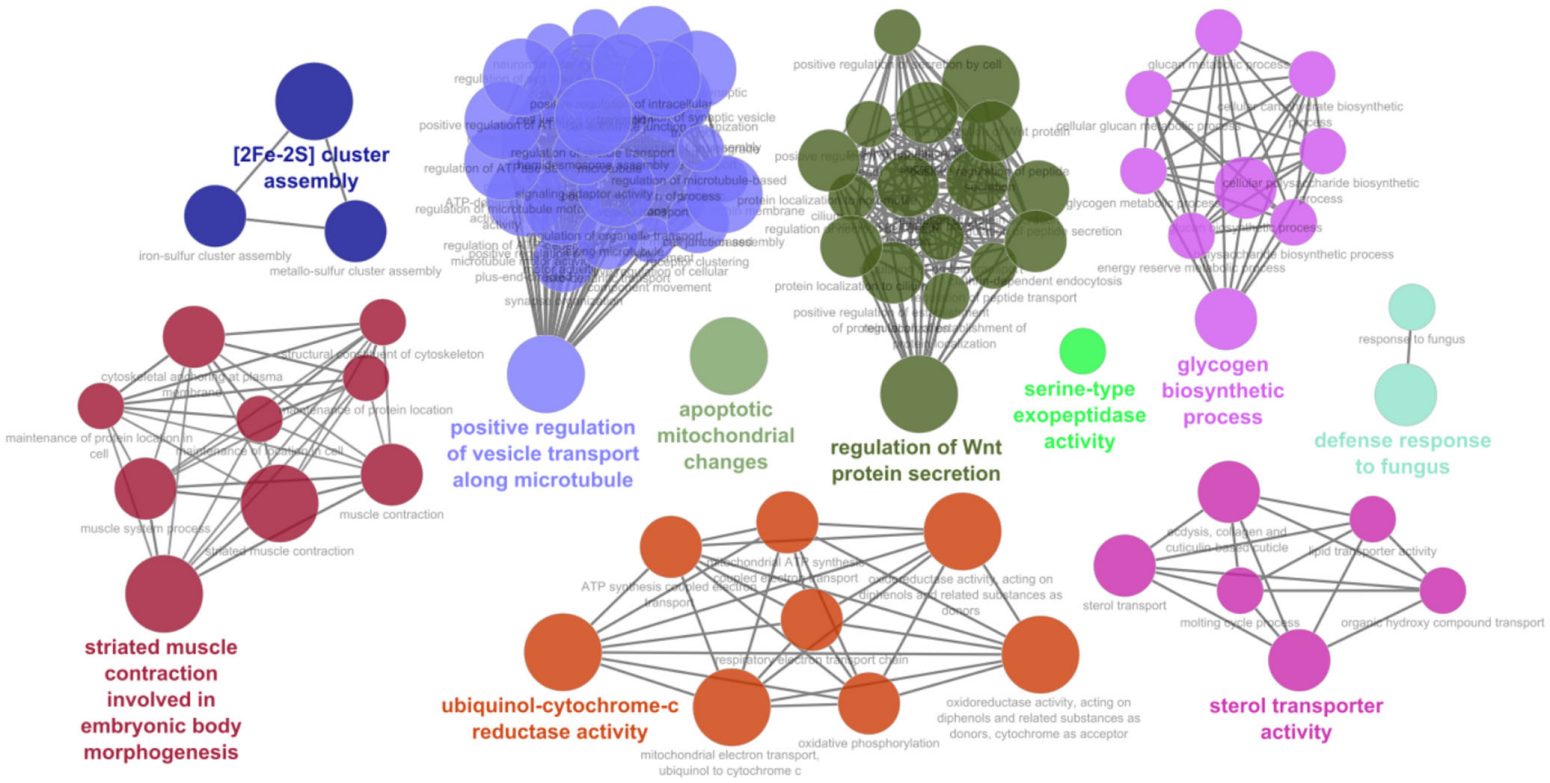
Biological processes and functional network clusters resulting from enrichment analysis carried out using the *S. lycopersicum* miRNA putatively targeting *M. incognita* mRNAs. The most relevant terms of the functional groups are highlighted.

When tomato miRNAs were evaluated against the nematode, the enrichment analysis of target genes resulted in BPs related to regulation of Wnt protein secretion, striate muscle contraction, sterol transported activity, ubiquinol-cytochrome-c reductase activity, [2Fe-2S] cluster assembly, glycogen biosynthetic process, defense response to fungus, serine-type exopeptidase, and apoptotic mitochondrial changes (**Figure 3**; **Supplementary Dataset 5**).

Among the most interesting examples investigated are sly-miR156, sly-miR166, and sly-miR319, given that they have been previously identified as highly abundant in roots during *M. incognita* infection (Kaur et al., 2017). The abundance of these miRNAs may favor their uptake in high amounts during feeding. **Table 2** presents the biological processes and putatively cross-kingdom targeted genes in relation to these three miRNAs. The data shows that sly-miR166(b,c) has the most abundant number of putative targets, distributed in GO terms related to response to stress and developmental processes. Although *M. incognita* genome has been sequenced (Abad et al., 2008), its annotation is not completed. To understand the roles of these putative targets we have looked at orthologues in related species. Among the genes involved in neuromuscular developmental processes, *rGCs* (guanylyl cyclases) plays a role in sensory processing (Maruyama, 2017), *lev-11* (LEVamisole resistant) encodes a conserved CUB-domain containing transmembrane protein (Gally et al., 2004), *snt-1* (SyNapTotagmin) functions as a Ca^2+^ sensor (Li L. et al., 2018), and *nep-2* (NEPrilysin metallopeptidase family) is a homolog of the extracellular peptidase neprilysin whose loss-of-function leads to movement anomalies (Soh et al., 2020). Impaired movement can be also caused by dysfunctions in the genes coding for proteins that are part of the collagen extracellular matrix. This may be the case of *dpy-17* (DumPY: shorter than wild-type), involved in cuticle development (Lang and Lundquist, 2021), and *emb-9* (abnormal EMBroygenesis), an alpha1(IV) collagen gene with embryo lethal effects when mutated (Guo et al., 1991; Li-Leger et al., 2021). Other putative target genes of sly-miR166 are involved in cell cycle regulation, like *cdc48* (Franz et al., 2014), *rpt-2* (Papaevgeniou and Chondrogianni, 2014), and *flub-2* (Haskell and Zinovyeva, 2021), nutrient availability (*rpb-7*, Collins et al., 2016), and gonad development (*gon-1*, Blelloch et al., 1999). Regarding sly-miR156a, this was predicted to target *dhc1*, encoding for the dynein heavy chain protein, and *smrc-1*, belonging to the Swi/snf chromatin remodeling complex. Dynein is an ATP-powered microtubule-based molecular motor, whose function includes the transport of cargo around the cell, while the loss-of-function of this gene inhibits apoptosis (Harders et al., 2018). The *smrc-1* gene is involved in the protection against DNA replication stress and its loss-of-function leads to the accumulation of chronic replication stress (Yang et al., 2019). Finally, sly-miR319a was predicted to target the *dlat-1* gene, encoding an enzyme with acetyltransferase activity; its lack of function leads to an early embryonic arrest (Li-Leger et al., 2021).

**Table 2:** List of representative *S. lycopersicum* miRNAs putatively targeting genes in *M. incognita* obtained from the cross-kingdom hybridization approach.

GO_ID	GO Term	*S. lycopersicum* miRNA	*M. incognita*Gene Accession	Orthologue Gene Name
**GO:0001101**	Response to acid chemical	sly-miR166b	Minc3s01925g27230 (Minc10092)	Guanylate cyclase (*M.hapla*, *G. pallida, C. elegans*)
			Minc3s03236g33246	F53F4.10 (*C. elegans*)
**GO:0002119**	Nematode larval development		Minc3s02352g29617	dpy-17, DumPY: shorter than wild-type (*C. elegans*)
			Minc3s02644g31015	dpy-17 (*C. elegans*)
**GO:0003006**	Developmental process involved in reproduction		Minc3s02193g28780	emb-9, abnormal EMBroygenesis (*C. elegans*)
			Minc3s00798g17488	gon-1,abnormal GONad development (*C. elegans*)
**GO:0002119**	Nematode larval development	sly-miR166c	Minc3s01495g24259	rpb-7, RNA Polymerase II (B) subunit (*C. elegans*)
**GO:0006950**	Response to stress		Minc3s01397g23468	cdc-48.1, cdc-48.2 (*C. elegans*)
**GO:0007275**	Multicellular organism development		Minc3s00400g11662	rpt-2,proteasome Regulatory Particle, ATPase-like (*C. elegans*)
**GO:0008219**	Cell death		Minc3s00015g00977	lev-10,(LEVamisole resistant) (*C. elegans*)
**GO:0009605**	Response to external stimulus		Minc3s00180g06927	Y54F10AR.1 (*C. elegans*)
			Minc3s02523g30444	Cre-snt-1 (*C. remanei*)
**GO:0032101**	Regulation of response to external stimulus		Minc3s00007g00476	Bma-lst-6 (*B. malayi*)
			Minc3s00643g15541	nep-2 NEPrilysin metallopeptidase family (*C. elegans*)
**GO:0031047**	Gene silencing by RNA		Minc3s00047g02560	fubl-2 FUBp (FUBP) Like (*C. elegans*)
**GO:0002119**	Nematode larval development	sly-miR156a	Minc3s01128g20986	dhc-1 Dynein Heavy Chain (*C. elegans*)
**GO:0000003**	Reproduction		Minc3s00513g13571	smrc-1, Swi/snf (SWI/SNF) related (*C. elegans*)
**GO:0007275**	Multicellular organism development	sly-miR319a	Minc3s00613g15138	dlat1, Dihydro- Lipoyllysine-residue AcetylTransferase (*C. elegans*)

Gene Ontology (GO) ID and terminology are provided along with gene names and corresponding accessions.

In the subsequent analysis, tomato miRNAs that share sequence homology with *M. incognita* miRNAs were analyzed via the seed region-based approach. Annotated orthologues of some predicted targets are collected in **Table 3**. Among the targeted biological processes, neurological development is much represented by putative targets such as *tgs-1* (trimethyl guanosine synthase), *gcy-9* (receptor-type guanylate cyclase), *mig-6* (abnormal cell MIGration, papilin), *kal-1* (human KALlmann syndrome homolog), as well as chemosensory genes like *Mi-odr-1* (Minc3s00015g01026, Minc3s00056g02910). A recent study indicated that *Mi-odr-1* is present in two copies in *M. incognita*, and was found to be expressed in the cell bodies of amphidal neurons and phasmids (Shivakumara et al., 2019). Silencing the *Mi-odr* and *Mi-gpa* genes could affect the nematode perception and infestation of the tomato root system (Li et al., 2022). Loss of *tgs-1* function in *C. elegans* leads to neurological phenotypes similar to those caused by the survival motor neuron (SMN) deficiency (Chen et al., 2022) whereas *gcy-9* mutants have different physiology in relation to adaptation and plasticity (Rossillo and Ringstad, 2020). The *C. elegans kal-1* gene affects epidermal morphogenesis by regulating the development of the substrate neuroblasts, and *kal-1* mutants show delayed migration of the ventral neuroblasts (Hudson et al., 2006). Loss-of-function mutants for the extracellular matrix molecule *mig-6* result in defects in dendrite formation (Ramirez-Suarez et al., 2019). Other important genes, like *unc-52* and *cbp*-1,2,3, are involved in larval development (Merz et al., 2003) and embryogenesis (Shi and Mello, 1998). Inhibition of these genes results in defects in myofilament assembly, larval movement deficiencies, or developmental arrest (Rogalski et al., 1995; Shi and Mello, 1998).

**Table 3:** List of some representative *S. lycopersicum* miRNAs putatively targeting genes in *M. incognita* obtained from the *seed region-based search* approach.

GO_ID	GO Term	*S. lycopersicum* miRNA	Min-miRNAs	*M. incognita* Gene Accession	Orthologue Gene Name
**GO:0050907**	Detection of chemical stimulus involved in sensory perception	sly-miR156a	min_miRNA51	Minc3s00025g01614 (Minc11367)	gcy-9, Receptor-type guanylate cyclase (*C. elegans*)
**GO:0007168**	Receptor guanylyl cyclase signaling pathway				
**GO:0007165**	Signal transduction			Minc3s02931g32115	T08G11.4, Trimethyl Guanosine Synthase homolog tgs-1 (*C. elegans)*
**GO:0008173**	RNA methyltransferase activity				
**GO:0031047**	Gene silencing by RNA				
**GO:0000902**	Cell morphogenesis	sly-miR319a	min_miRNA306, NOVEL-5	Minc3s01898g27043 (Minc13237)	mig-6, abnormal cell MIGration (*C. elegans*)
**GO:0040002**	Collagen and cuticulin-based cuticle development				
**GO:0002119**	Nematode larval development			Minc3s01574g24825 (Minc12944)	unc-79, UNCoordinated, (*C. elegans*)
**GO:0042493**	Response to drug				
**GO:0007044**	Cell-substrate junction assembly	sly-miR169f, sly_miRNA3291	NOVEL-40, min_miRNA37	Minc3s00001g00015 (Minc00111)	unc-52, UNCoordinated (*C. elegans*)
**GO:0006941**	Striated muscle contraction			Minc3s00053g0281 (Minc11499)	Cbr-unc-52 (*C.briggsae*)
**GO:0009792**	Embryo development ending in birth or egg hatching	sly-miR169e-3p	miR-72	Minc3s00145g05964 (Minc02858)	kal-1, human KALlmann syndrome homolog (*C. elegans*)
**GO:0048730**	Epidermis, morphogenesis				
**GO:0003724**	RNA helicase activity			Minc3s02703g31258	ddx-27, DEAD boX helicase homolog (*C. elegans*)
**GO:0016887**	ATP hydrolysis activity				
**GO:0007275**	Multicellular organism development		miR-72_1	Minc3s01556g24691	T09B9.4 (*C. elegans*)
**GO:0009790**	Embryo development			Minc3s00060g03099	cbp-3, cbp-2, CBP/p300 homolog (*C. elegans*)
**GO:0006915**	Apoptotic process				
**GO:0007018**	Microtubule-based movement	sly_miRNA4126	min_miRNA32	Minc3s01128g20986 (Minc02896)	dhc-1, Dynein Heavy Chain (*C. elegans*)
**GO:0005524**	ATP binding				

Gene Ontology (GO) ID and terminology are provided along with sly-miRNAs, min-miRNAs, M. incognita accessions and orthologous from related species (e.g., Caenorhabditis elegans, Caenorhabditis briggsae).

Hence, most *M. incognita* genes putatively targeted by sly-miRNAs have important roles in nematode development, leading to adverse effects on digestion, mobility, and reproduction, often with lethal outcomes. This finding is of utmost importance for the agricultural sector in view of developing plant miRNA-based technologies to control nematode diffusion.

When considering the use of different computational methods to predict miRNA targets in a cross-kingdom manner, such as the ones used in this work, it is important to underline that these rely on different assumptions on targeting rules and are still necessary to face our currently limited knowledge in miRNA trans-species interactions. Experimental confirmations are therefore needed to understand the actual targeting roles of the illustrated miRNAs. The availability of these computational methods is however useful to guide researchers in the selection of miRNA candidates for further investigation, despite the target prediction algorithm outcomes could be different due to the different underlying assumptions.

### 3.4 *In vitro* experimental validation of tomato miRNAs influencing *M. incognita* infection

To investigate the hypothesized cross-kingdom miRNAs transfer along with its potential to control the RKN infection, an *in vitro* experimental system was designed based on soaking assay, nematode larvae phenotyping, and gene expression profiling. To this purpose, J2 larvae were fed with solutions containing tomato miRNAs (sly-miR166b, sly-miR156a, sly-miR169f) selected from the bioinformatics data. The sly-miR166b - *Minc3s01925g27230* (formerly named *Minc10092*) pair was chosen from the cross-kingdom hybridization approach (**Table 2**). No *C. elegans* homolog was found for this gene but a protein orthologue was identified as guanylate cyclase (UniProtKB/TrEMBL accession A0A1I8B0V8_MELHA) in *M. hapla.* The sly-miRNA156a - *Minc3s00025g01614* (formerly named *Minc11367*) pair was selected from the seed-based approach (**Table 3**). This miRNA has a complete seed-region homology with min_miRNA51 targeting the *Minc11367* gene, as shown in **Supplementary Figure 1**. In plants, it is well known that miR156 targets the SQUAMOSA PROMOTER BINDING PROTEIN-LIKE (SPL) transcription factor, controlling genes involved in the regulation of reactive oxygen species (ROS) (Yin et al., 2019). In the RKN trans-kingdom approach, this sly-miRNA putative target was predicted as the *C. elegans* homologous *gcy-9* (guanylyl cyclase, WBGene00001536). A homologue of this gene was also identified in the parasitic nematode *Haemonchus contortus*, a close relative of *M. incognita* (Winter et al., 2012). Lastly, sly-miR169f was predicted to target *Minc3s00001g00015* (formerly named *Minc00111*, **Supplementary Figure 2**), an orthologue of the *C. elegans unc-52* (WBGene00006787) gene. In the GO analysis, this accession was related to functions connected to cell junction organization or muscle system.

**Figure 4:**
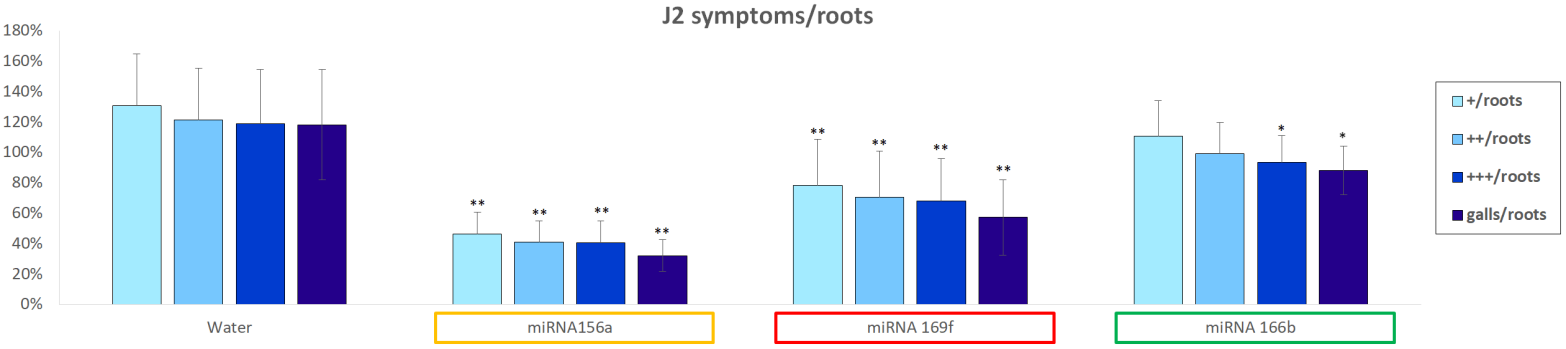
*In vitro* assay regarding soaked sly-miRNAs J2 larvae and susceptibility of the tomato root infections. The symptoms of infection (normalized with respect to the number of roots) with increasing apex enlargement, are categorized as “+”, “++”, “+++” and “galls”. Data represents percentages expressed compared to the soaked J2s control solution, expressed as mean ± SD of three independent experiments for each plant/miRNA. Statistical differences, in terms of Student’s *t*-test, are given (*P ≤ 0.05; **P ≤ 0.01). FDR, False Discovery Rate.

Following the selection of tomato miRNAs to be tested in the nematode system, an *in vitro* soaking assay was performed as described in Section 2.4. The motility and vitality of the J2 larvae were observed and compared with those of larvae kept in the control solution. Subsequently, these larvae were used to infect tomato seedlings. **Figure 4** shows the effects of the infection symptoms classified as “+”, “++”, “+++”, along with galls formation on the roots. The values represent the percentage of symptoms normalized to the number of roots where the phenomena were observed. When the effects of the control solution and sly-miRNA156a soaked J2 larvae were compared, the best statistically significant reduction was detected in terms of gall formation. The amount of “+/roots”, “++/roots” and “+++/roots” was also reduced up to 1/3, while the number of “galls/roots” was reduced up to 1/4. A significant reduction of the same parameters was observed also for the J2s soaked with sly-miR169f, where root enlargements (“+/roots”, “++/roots”, “+++/roots”) were reduced up to 60% compared to control, and the number of “galls/roots” was halved. In the case of sly-miR166b, the observed reduction was less prominent, ranging from 15% (“+/roots”) to 25% (“galls/roots”).

**Figure 5:**
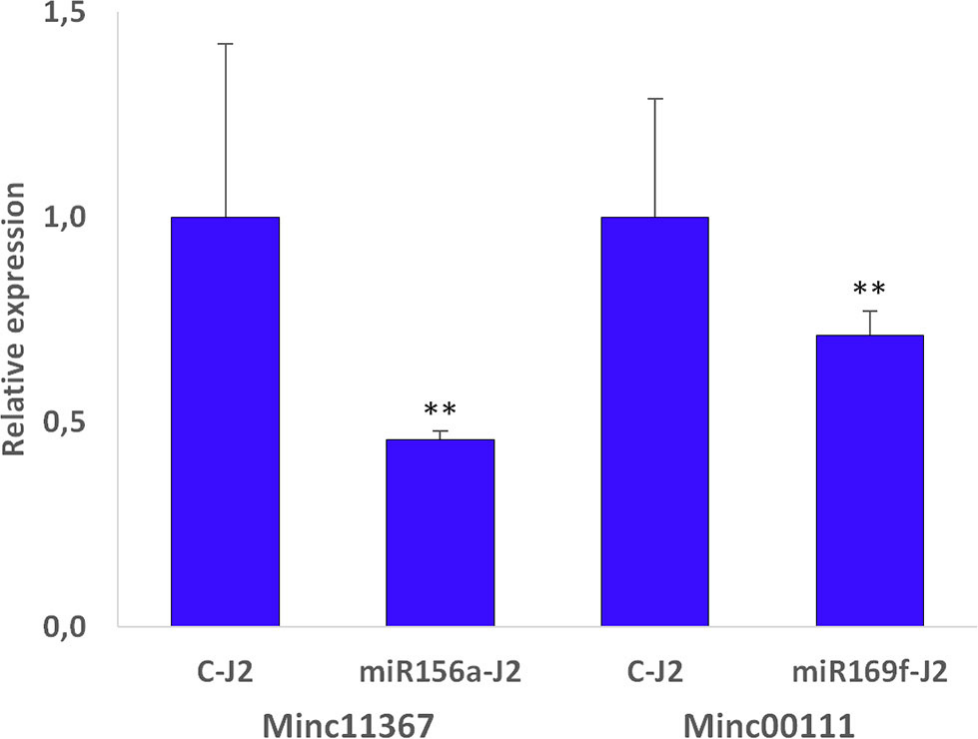
Relative expression data in miRNA156a and miRNA169f soaked J2s relative to control (C-J2). Transcript levels for *Minc11367* (Minc3s00025g01614) and *Minc00111* (Minc3s00001g00015) were measured by qRT-PCR after soaking treatment and compared to their transcript level in control samples. Data are shown as mean ± SD from two independent replicates. Statistical differences, in terms of Student’s *t*-test, are given (**P ≤ 0.01).

Because the most prominent reduction of the galling process was observed for the sly-miRNA156a and sly-miRNA169f soaked larvae, these were selected for further molecular analysis. Quantitative RealTime-PCR was employed to measure the relative expression of the *Minc11367* (Minc3s00025g01614) and *Minc00111*(Minc3s00001g00015) genes, putatively targeted by these miRNAs. The obtained data show a downregulation of 54% in the miR156a-J2 and 29% in the miR169f-J2 treated larvae compared to the control (**Figure 5**). This result indirectly indicates that, in the *in vitro* experimental setup applied in this study, sly-miRNA156a and sly-miRNA169f have a cross-kingdom influence on the expression of *Minc11367* and *Minc00111* nematode genes.

**Figure 6:**
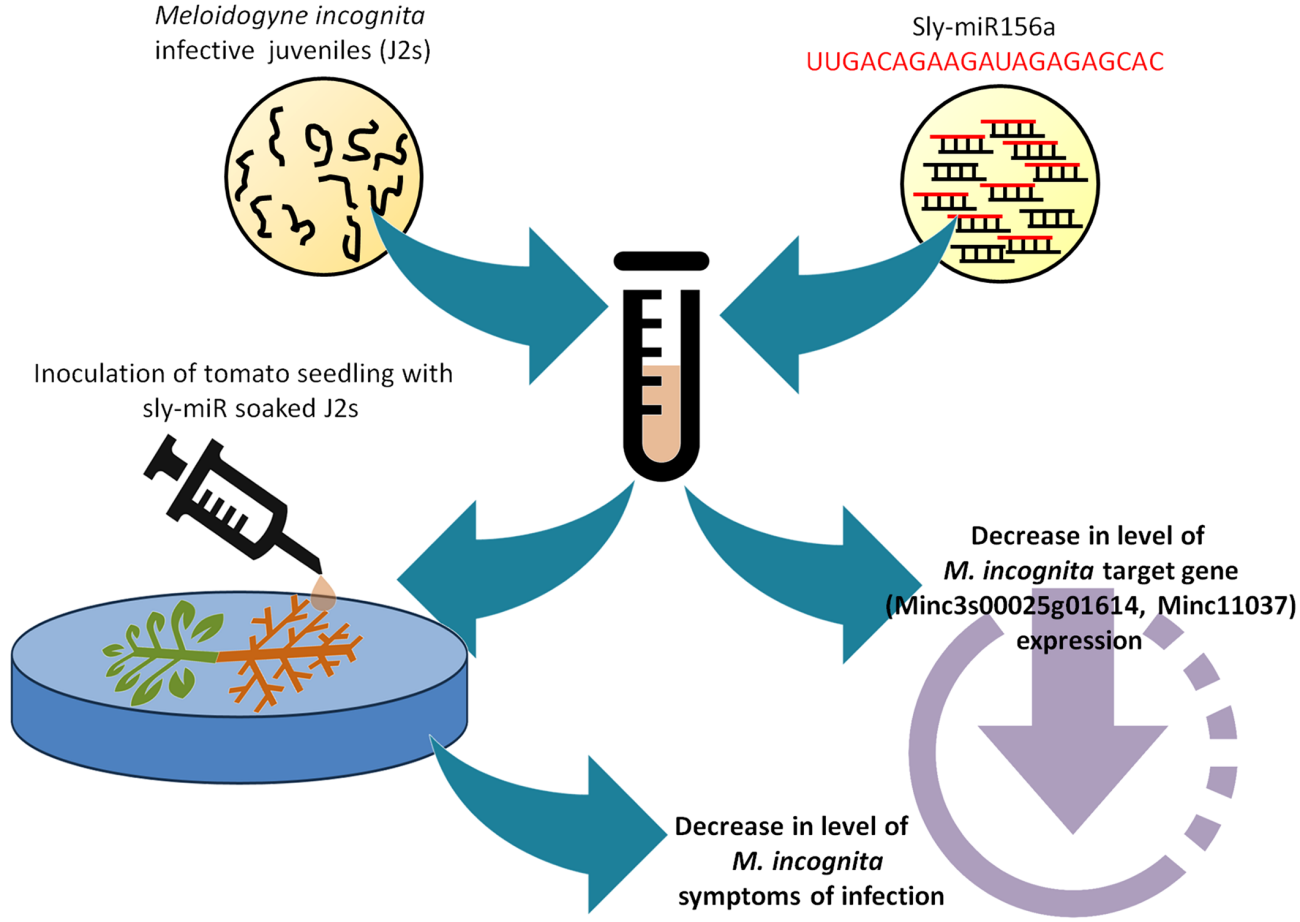
Schematic representation of the *in vitro* feeding assay showing the Sly-miRNAs soaking experiments performed on J2 larvae. The inoculation of tomato seedlings with sly-miR caused a decrease in the *M. incognita* infection symptoms and downregulation of putative cross-kingdom targets.

To summarize these findings, **Figure 6** shows a schematic representation of how the *in vitro* sly-miRNAs soaking experiments performed on J2 larvae led to a decrease in the *M. incognita* infection symptoms along with the downregulation of putative cross-kingdom targets. To our knowledge, this is the first evidence showing that tomato miRNAs can be used to alter the infection ability of *M. incognita* larvae. Other studies related to the trans-kingdom transfer of tomato miRNAs to pathogens focused mainly on the *Botrytis cinerea* fungal infection. For instance, sly-miR1001 was shown to inhibit fungal virulence and conidiospore germination by targeting genes encoding for an ATP-dependent metallopeptidase and a cysteine-type endopeptidase (Meng et al., 2020). More recently, a genome-wide study identified multiple sRNAs and miRNAs with antifungal properties (Wu et al., 2023). The same study demonstrated that exogenous application of sly-miR396a was able to suppress the virulence of *B. cinerea*. Therefore, the data provided in our study together with other experimental evidence form the literature, evidence that tomato miRNAs can be effectively used to limit plant pathogen infection. However, given that most studies provide *in vitro* evidence to support this fact, in the future it would be required to further investigate how this transfer occurs and how is it conditioned by concentration and bioavailability in an *in vivo* system.

## 4 Conclusions

Following the increasing availability of omics data, bioinformatic studies are providing powerful tools to aid in setting up pertinent experimental designs based on preliminary predictions. This applies to miRNA-target interactions (both intra- and inter-specific) since different tools are available for target predictability based on sequence complementarity or hybridization energy. In this work, bidirectional bioinformatics analyses were conducted to uncover the potential miRNA-dependent cross-kingdom interactions between tomato and the phytoparasitic nematode *M. incognita* in terms of putative repressed genes and related biological processes. The obtained results are compatible with the host-parasite interactions between tomato and RKNs, suggesting that exogenous miRNAs may play a role in such processes. Although bioinformatics provides solid grounds to formulate hypotheses, some limitations are present considering the use of different computational methods, target prediction algorithms, and the lack of general guidelines applicable specifically for cross-kingdom predictions. Therefore, an *in vitro* experimental system was developed to support the potential cross-kingdom miRNAs effect during the *S. lycopersicum* - *M. incognita* interaction. The presented data evidence that the administration of sly-miR156a and sly-miR169f to J2s larvae was able to significantly lower root infection. This was also coupled with the downregulation of predicted cross-kingdom targets, *Minc11367* and *Minc00111*. Thus, the current study expands the knowledge on the host-parasite interactions between tomato and RKNs paving the way for future application of exogenous miRNAs as tools to control *M. incognita* infection. However, this is still preliminary work and further analyses should be taken into account to understand the highly complex *in vivo* mechanism of plant miRNA-mediated gene control in the context of nematode research.

## Data availability statement

The original contributions presented in the study are included in the article/**Supplementary Material**. Further inquiries can be directed to the corresponding authors.

## Ethics statement

The manuscript presents research on animals that do not require ethical approval for their study.

## Author contributions

PL: Writing – original draft, Writing – review & editing, Conceptualization, Funding acquisition, Data curation. DD: Writing – review & editing, Software. DM: Writing – review & editing, Software. PC: Writing – review & editing, Software. LP: Writing – original draft, Writing – review & editing, Conceptualization, Data curation. AM: Writing – original draft, Writing – review & editing, Conceptualization, Data curation.

## Supplementary material

Supplementary Data Sheet 1 Complete dataset regarding cross-kingdom prediction based on *M. incognita* miRNAs putatively targeting genes in *S. lycopersicum.* Information regarding miRNA and target sequence, perfect seed complementarity (no GU), seed complementarity (with GU), percentage (%) of the match without seed and GU, % match without seed but with GU, and multiplicity, are provided.

Supplementary Data Sheet 2 Complete dataset generated using the *cross-kingdom hybridization* approach regarding *S. lycopersicum* miRNAs putatively targeting genes in *M. incognita.* Information regarding Minimum Free Energy (MFE), miRNA and target sequence, perfect seed complementarity (no GU), seed complementarity (with GU), percentage (%) of the match without seed and GU, % match without seed but with GU, multiplicity, and 3’ÚTR targeting, are provided.

Supplementary Data Sheet 3 Complete dataset using the *seed region-based search* approach regarding *S. lycopersicum* miRNAs putatively targeting genes in *M. incognita.* Information regarding Minimum Free Energy (MFE), miRNA and target sequence, perfect seed complementarity (no GU), seed complementarity (with GU), percentage (%) of the match without seed and GU, % match without seed but with GU, multiplicity, and 3’ÚTR targeting, are provided.

Supplementary Data Sheet 4 List of enriched *S. lycopersicum* Biological Process GO terms, with the relevant terms of the functionally grouped term networks provided by ClueGO shown in bold. Genes relative to each term and the miRNAs targeting each gene are shown.

Supplementary Data Sheet 5 List of enriched *M. incognita* Biological Process GO terms, with the relevant terms of the functionally grouped term networks provided by ClueGO shown in bold. Genes relative to each term and the miRNAs targeting each gene are given.
