# Supplementary material for: Exploring the putative microRNAs cross-kingdom transfer in Solanum lycopersicum-Meloidogyne incognita interactions
Source: Front Plant Sci. 2024 May 8;15:1383986. doi: 10.3389/fpls.2024.1383986 (PMC11114104; doi:10.3389/fpls.2024.1383986)
Supplement: Supplementary file 1 [file DataSheet_1.docx]

Supplementary Material

**Exploring the Putative MicroRNAs Cross-Kingdom Transfer in *Solanum lycopersicum* -*Meloidogyne incognita* Interactions**

**Paola Leonetti^1,*^, Debora Dallera****^2^, Davide De Marchi^2^, Pamela Candito^2^, Lorenzo Pasotti^2,*^, Anca Macovei^3,*^**

^1^Institute for Sustainable Plant Protection of the National Research Council, Research Unit of Bari, Bari, Italy

^2^Laboratory of Bioinformatics, Mathematical Modelling, and Synthetic Biology, Department of Electrical, Computer and Biomedical Engineering - Centre for Health Technology, University of Pavia, Pavia, Italy

^3^Plant Biotechnology Laboratory, Department of Biology and Biotechnology “L. Spallanzani”, University of Pavia, Pavia, Italy

*** Correspondence:**

Paola Leonetti, [paola.leonetti@cnr.it](mailto:paola.leonetti@cnr.it)
Lorenzo Pasotti, [lorenzo.pasotti@unipv.it](mailto:lorenzo.pasotti@unipv.it)

Anca Macovei, [anca.macovei@unipv.it](mailto:anca.macovei@unipv.it)

**Supplementary Table 1.** List of oligonucleotide sequences used for the qRT-PCR reactions.

| **Gene** | **Wormbase Accession** | **FW sequence (5’-3’)** | **REV sequence (5’-3’)** |
| --- | --- | --- | --- |
| *Minc11367* | Minc3s00025g01614 | CCAGACGAAATAATGATGGTCA | TATCGCCAAAAAGGCAATAG |
| *Minc00111* | Minc3s00001g00015 | CTGTCAATCTGGGACGTTCT | TCCTTGGTGATCGTGTTCAG |
| *18SribRNA* (reference) | Minc3s09153g42974 | ACCGTGGCCAGACAAACTAC | GATCGCTAGTTGGCATCGTT |

**
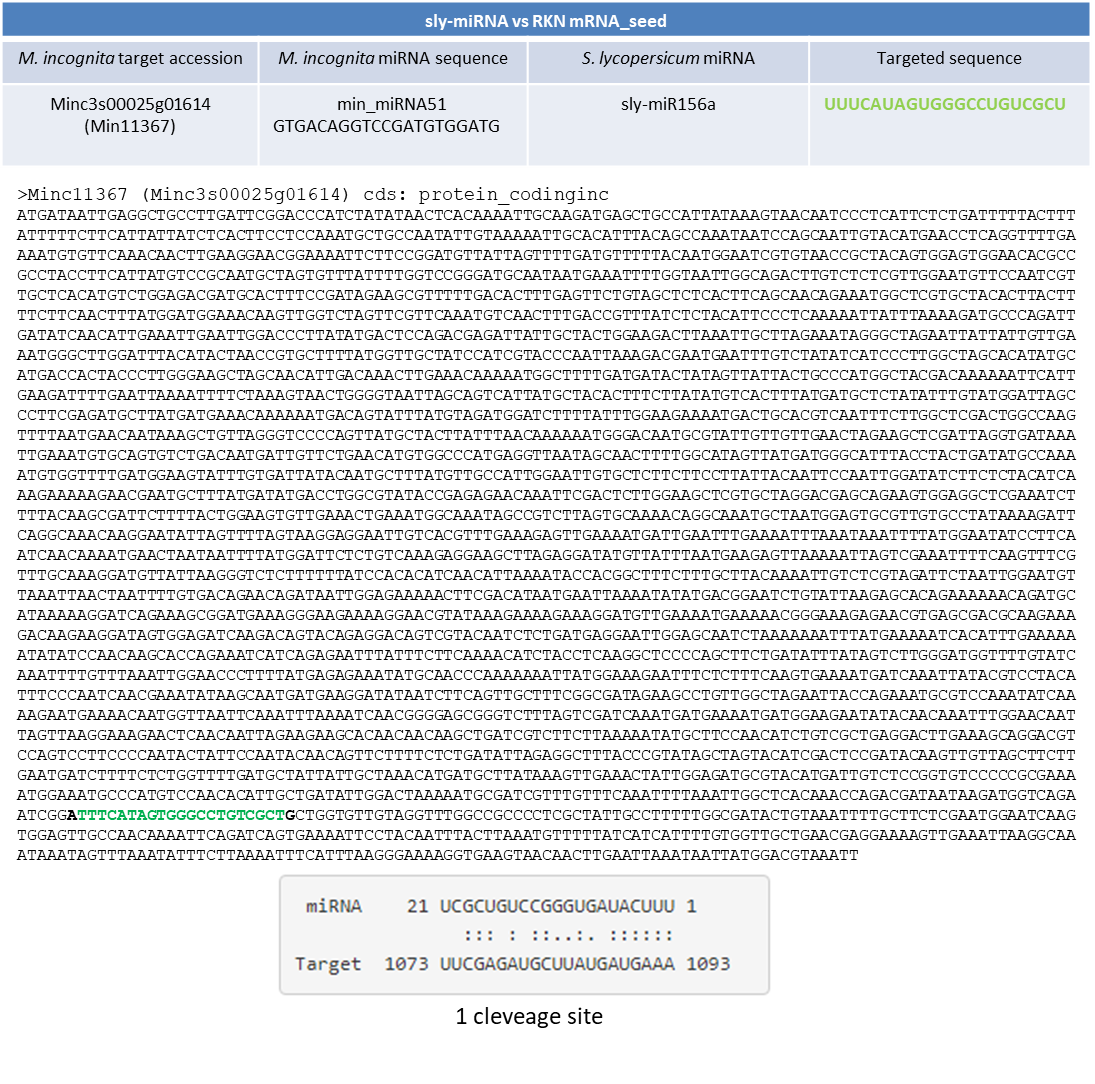
**

**Supplementary Figure 1.** Minc11367 (Minc3s00025g01614) sequence and the cleavage site as revealed by the psRNATarget tool. The target prediction relates with data available in the Supplementary Dataset 3 and is summarized in the figure. In green the *M. incognita* sequence used for designing primers for the qRT-PCR analysis.

**
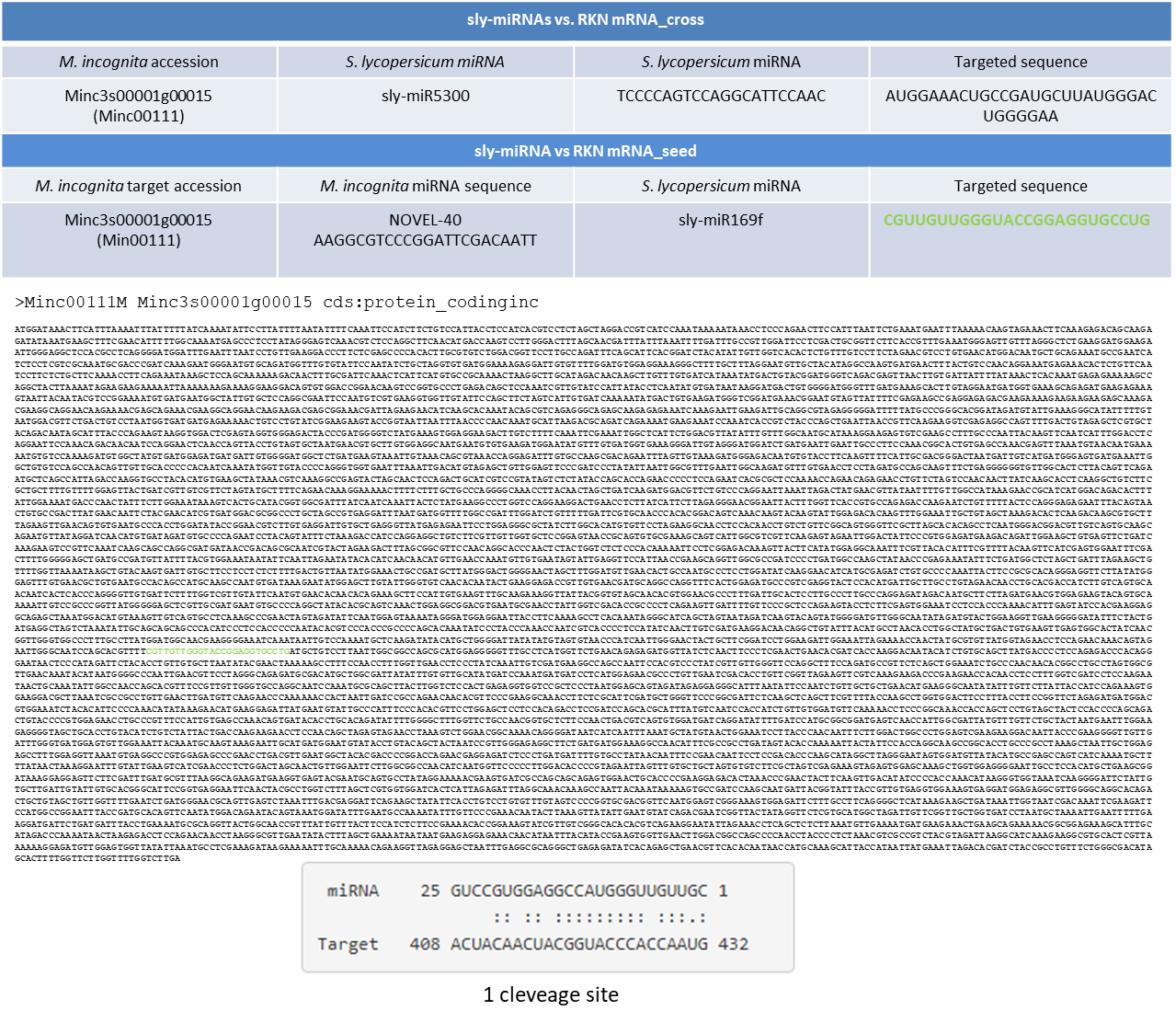
**

**Supplementary Figure 2.** Minc00111 (Minc3s00001g00015) sequence and the cleavage site as revealed by the psRNATarget tool. The target prediction relates with data available in the Supplementary Dataset 2 and Supplementary Dataset 3 and is summarized in the figure. In green the *M. incognita* sequence used for designing primers for the qRT-PCR analysis.
